# Histological evaluation following treatment of recession-type defects with coronally advanced flap and a novel human recombinant amelogenin

**DOI:** 10.1007/s00784-023-05123-x

**Published:** 2023-07-08

**Authors:** Tali Chackartchi, Dieter D. Bosshardt, Jean-Claude Imber, Alexandra Stähli, Hagit Sacks, Katalin Nagy, Anton Sculean

**Affiliations:** 1 Department of Periodontology, Hadassah Medical Center, Faculty of Dental Medicine, Hebrew University of Jerusalem, Jerusalem, Israel; 2 Robert K. Schenk Laboratory of Oral Histology, Department of Periodontology, School of Dental Medicine, University of Bern, Bern, Switzerland; 3 Department of Periodontology, School of Dental Medicine, University of Bern, Freiburgstrasse 7, 3010 Bern, Switzerland; 4 VP R&D Prudentix. Ltd., Lod, Israel; 5 Department of Oral Surgery, Faculty of Dentistry, University of Szeged, Szeged, Hungary

**Keywords:** Recombinant Amelogenin, Periodontal regeneration, Gingival recessions, Periodontal wound healing

## Abstract

**Objectives:**

To histologically evaluate the effects of a novel human recombinant amelogenin (rAmelX) on periodontal wound healing / regeneration in recession-type defects.

**Materials and methods:**

A total of 17 gingival recession-type defects were surgically created in the maxilla of three minipigs. The defects were randomly treated with a coronally advanced flap (CAF) and either rAmelX (test), or a CAF and placebo (control). At three months following reconstructive surgery, the animals were euthanized, and the healing outcomes histologically evaluated.

**Results:**

The test group yielded statistically significantly (*p* = 0.047) greater formation of cementum with inserting collagen fibers compared with the control group (i.e., 4.38 mm ± 0.36 mm vs. 3.48 mm ± 1.13 mm). Bone formation measured 2.15 mm ± 0.8 mm in the test group and 2.24 mm ± 1.23 mm in the control group, respectively, without a statistically significant difference (*p* = 0.94).

**Conclusions:**

The present data have provided for the first-time evidence for the potential of rAmelX to promote regeneration of periodontal ligament and root cementum in recession-type defects, thus warranting further preclinical and clinical testing.

**Clinical relevance:**

The present results set the basis for the potential clinical application of rAmelX in reconstructive periodontal surgery.

## Introduction

The apical displacement of the gingival margin results in root exposure to the oral cavity, which might cause aesthetic concerns, root sensitivity and often impair adequate oral hygiene, which in turn, may lead to the development of gingivitis, further attachment loss or root caries [1]. Over the last decades, various surgical procedures have been proposed to predictably cover gingival recessions [2, 3]. Systematic reviews on the efficacy of these surgical procedures reported that the use of an enamel matrix derivative (EMD) applied onto the root surfaces in conjunction with a coronally advanced flap (CAF) increased the probability of achieving complete root coverage [4, 5]. Histologic data from animals and humans have provided robust evidence on the potential biological effects of EMD to facilitate periodontal regeneration (i.e. formation of root cementum, periodontal ligament, and alveolar bone) in recession-type defects [6–8]. Moreover, the additional application of EMD to connective tissue grafts in conjunction with CAF has been shown to promote periodontal regeneration, thus facilitating coverage of gingival recessions [9].

It is well known, that amelogenins are the major enamel proteins produced by ameloblasts during amelogenesis. They comprise 90% of the developing extracellular enamel matrix proteins [10, 11], and are considered accountable for the biological effect of EMD in periodontal wound healing and regeneration [12].

An important aspect, however, is the origin of EMD, which is extracted from the enamel matrix of developing pig teeth. This has obvious limitations in terms of the amount that can be produced and the variability in the amelogenin content. Moreover, the production of anti-EMD antibodies in the host has been reported [13]. To overcome these shortcomings, substantial efforts have been made to produce amelogenin in a recombinant way [14].

A novel human recombinant amelogenin (rAmelX) has been recently introduced in regenerative periodontal therapy [15]. However, at present, the potential effect of rAmelX on periodontal wound healing/regeneration in recession-type defects is unknown.

Therefore, the aim of the present pilot study was to histologically evaluate the effects of rAmelX on periodontal wound healing/regeneration in recession-type defects.

The study hypothesis was that treatment of recession-type defects with CAF and rAmelX used in conjunction with its carrier, will promote periodontal regeneration (i.e., regeneration of cementum, periodontal ligament, and alveolar bone) to a higher extent than treatment with CAF and carrier.

## Materials and methods

### Animals

Three healthy inbred Sinclair miniature female pigs, 18 months old and weighing 55–60 kg, were used in this study. The animals were housed and monitored daily for the duration of the study in the Institute of Animal Research in Kibbutz Lahav, Negev, Israel. They were kept at 20 °C to 26 °C, at a relative humidity of 30% to 70%, and a 12-h light/dark cycle. A standard diet (soft food soaked in water) expanded for the minipigs was provided to each animal daily (AMBAR, Feed mill, Granot M.P. Heffer 3881100, Israel), and water was available ad libitum. All procedures during the in-life phase were approved by the ethical committee of the Animal Research Center (No.IL-20–3-93). This study was conducted in accordance with the ARRIVE guidelines for preclinical animal studies and the Israeli National Council of Animal Experimentation.

### Surgical protocol

All surgical procedures were performed under general and local anesthesia using aseptic routines by one experienced surgeon (TC). Prior to the surgeries, 2 mg/kg xylazin (Anased®, AKORN, USA) + 10 mg/kg ketamine (Clorkeam®, Vetoquinol, France) were administered by intramuscular injection. Anesthesia was induced by inhalation of 3% isoflurane (Piramal critical care, USA) via a mask and intravenous administration of 10 mg diazepam per animal (Diazepam-Ratiopharm®, Ratiopharm, Germany). During surgeries, the animals were intubated, and anesthesia was maintained by administration of 1–3% isoflurane. Prior to surgery, a 1.8 ml mixture of local anesthesia (10 ml lidocaine 20 mg/ml, Esracain®, Rafa lab, Israel + 0.125 ml adrenaline 1 mg/ml, Adrenalina®, Galenica Senese, Italy) was administered by infiltration at the surgical site.

**Fig. 1:**
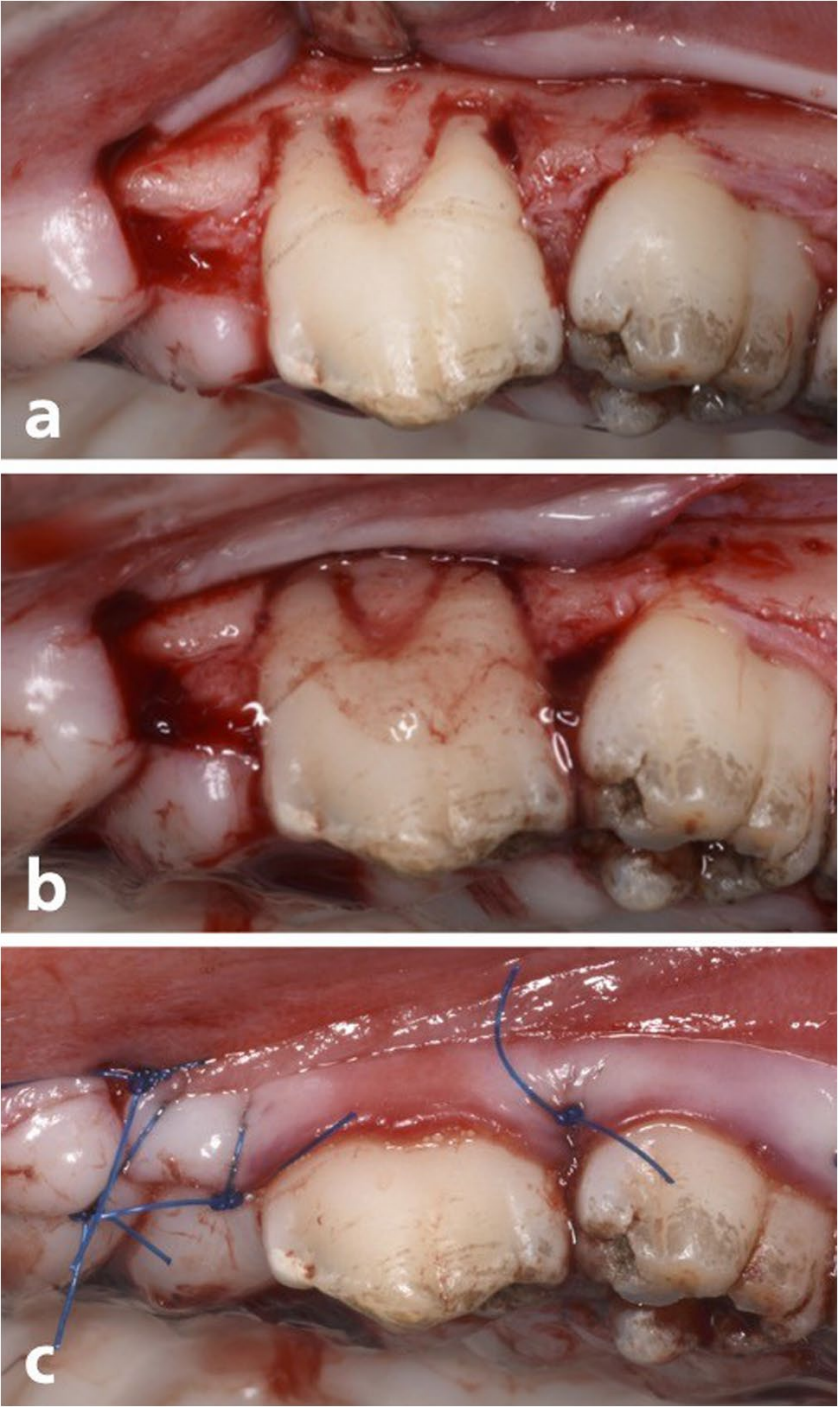
Surgical pictures illustrating the procedure in the test group. **(a)** After Flap elevation and defect creation **(b)** application of the biomaterial (rAmelX) **(c)** after wound closure

After the induction of anesthesia, recession defects were surgically created bilaterally in the maxilla of each animal according to a modification of a method described previously by Nunez et al. [16]. Two vertical releasing incisions were made on the mesio-buccal aspect of PM 2 and on the disto-buccal aspect of PM 4. A muco-periosteal flap was elevated exposing the buccal alveolar bone. Dehiscence defects were created on PM 2 and PM 4 unless there were difficulties to access PM4. In these cases, lesions were created on PM2 and PM3. The defects measured 5 mm in depth from the cemento-enamel junction and 3 mm in width (Fig. 1a). Root surfaces were thoroughly scaled and planed using hand instruments (Gracey curettes) to completely remove the root cementum and periodontal ligament. A notch was prepared (notch A) with a small diamond round bur on the root surface at the bottom of the defect under irrigation with sterile saline. The maxillary recession defects were randomly assigned to one of the following treatments:

- Test group: CAF + rAmelX—(Recombigain, 0.5 mg/ml rAmelX plus carrier) (Prudentix Ltd, Israel)
- Control group: CAF + carrier only (Proprietary Formulation) – (i.e., Recombigain placebo)

Recombigain is a proprietary thermo-sensitive gel-based composition of X- linked human recombinant amelogenin. The rAmelX protein was manufactured using an e-coli expression system. The thermos-sensitive gelling agent in the composition is poloxamer. The poloxamer was used as a control. According to the allocated group procedure, the material was administered on the exposed root surface (Fig. 1b).

The control and test material were prepared as a transparent gel, in an un-marked syringe. The surgical assistant was dispensing the assigned material for each lesion, whereas the surgeon was blind to the material in use.

Finally, the flaps were sutured (Fig. 1c) and stabilized using marginal direct sutures to adapt the flap to the neck of the teeth at the inter-dental papillae, using monofilament 4–0 non-resorbable nylon sutures (ETHILON™).

### Post-surgical protocol

Post-surgery pain was controlled with Buprenorphine (0.0.5 mg/kg, IM) for 7 days and Carprofen (2–4 mg/kg, IM) for 7 days. Antibiotics (Amoxiclav, 1 ml/20 kg, SID, SC) was administrated for 7 days. The animals were monitored twice daily by a technician and once weekly by a veterinarian for the health symptomatology including animal behavior, food and water uptake, and examination of the oral cavity. The animals were fed Ensure (Abbot) for 5 days post operation and thereafter a soft-pellet diet for another week. The remaining dentition received oral prophylaxis using gauzes soaked in a 0.2% chlorhexidine solution.

### Samples retrieval and histologic preparation

Three months after surgery, the animals were euthanized by Isoflurane inhalation, sedated with Pental (8 ml, IV) and KCl (20 ml, IV). The maxillae were removed, and individual bone blocks containing the implanted biomaterials and the surrounding soft and hard tissues were taken and subsequently fixed with 10% neutral-buffered formalin.

After fixation, the specimens were rinsed in tap water, dehydrated in ascending concentrations of ethanol, infiltrated, and embedded in methylmethacrylate. After polymerization, the embedded blocks were trimmed and cut along the root axis of each involved tooth on a bucco-palatal plane. Ground sections were produced using a high-precision, slow-speed diamond disk with a coolant (Varicut® VC-50; Leco, Munich, Germany). After mounting onto acrylic glass slides, the sections were ground to a final thickness of 100 μm with a specially designed grinding machine (Knuth-Rotor- 3; Struers, Rodovre/Copenhagen, Denmark) and superficially stained with toluidine blue/McNeal combined with basic fuchsin. The two central-most ground sections per defect were chosen for descriptive and histometric analyses. High-resolution photography was performed using a digital camera (AxioCam MRc; Carl Zeiss, Oberkochen, Germany) connected to a light microscope (Axio Imager M2; Carl Zeiss).

### Histometric analysis

The central sections (displaying clearly distinguishable apical and coronal notches and depicting the central location within the defect area) were chosen for the histometric analysis. Regions of interest were digitalized with a computer connected to a light microscope (Axio Imager M2; Carl Zeiss).

**Fig. 2:**
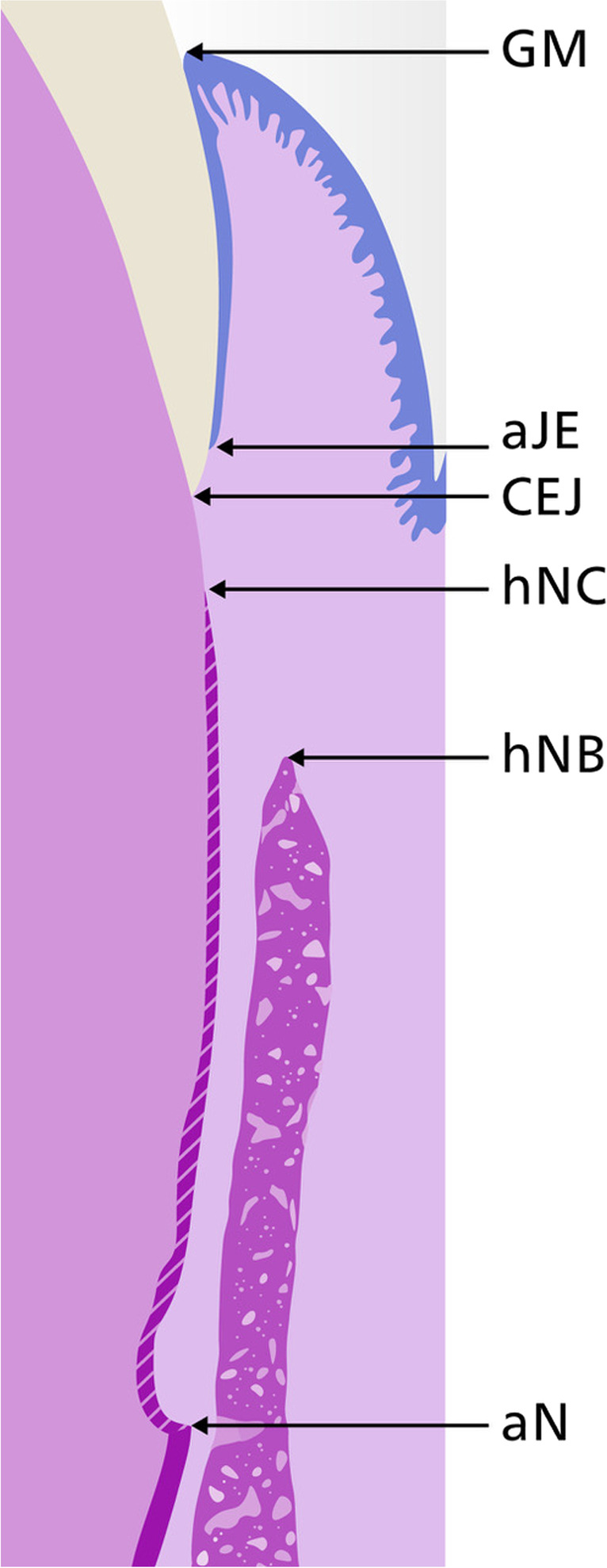
Histometrical landmarks. aJE, apical end of junctional epithelium; aN, apical notch; CEJ, Cemento-enamel junction (CEJ); GM, Gingival margin; hNB, highest point of new bone; hNC, highest point of new cementum

All necessary histological landmarks (Fig. 2) were identified by two experienced investigators (DDB + JCI):

- Gingival margin (GM)
- Cemento-enamel junction (CEJ)
- Apical end of the junctional epithelium (aJE)
- Apical notch (aN)
- Highest point of new cementum (hNC)
- Highest point of new bone (hNB)

After identification, the following histometric measurements were performed by a single examiner (JCI):

- Defect height: aN—CEJ
- Junctional epithelium (JE) height including sulcus depth: aJE – GM
- Connective tissue adhesion (CT): hNC – aJE
- New bone height: aN—hNB
- New cementum height: aN—hNC

### Statistical analysis

To assess the difference between the two groups, 2 sections per defect were analyzed. Intergroup differences among the defects were compared utilizing the non-parametric Wilcoxon signed-rank test via Graph Prism 9 (GraphPad Software, La Jolla, CA, USA). Data are tabulated as mean and standard deviation, median, minima and maxima, as well percentile ranks. *P* < 0.05 is considered statistically significant. Sample size calculation was based on an equal variability assumption, a two-tailed alpha error of 0.05 and a 90%-power resulting in a minimum of 7 defects per group.

Sample processing and statistical analysis were conducted in codded groups (i.e. blinded to the test and control).

## Results

### Clinical observations

The postoperative healing was uneventful at all sites. There were no signs of impaired health including animal behavior as well as food and water uptake, which were recorded to be normal. At the surgical site, no adverse reactions such as suppuration, abscess formation, or increased tooth mobility were detected throughout the entire experimental period. Three defects could be created on every hemi-maxilla, excepting one side where only two defects could be created, due to a natural occurring dehiscence defect. Consequently, a total of 17 defects were treated (i.e., 9 in the test and 8 in the control group).

### Descriptive histology

**Fig. 3:**
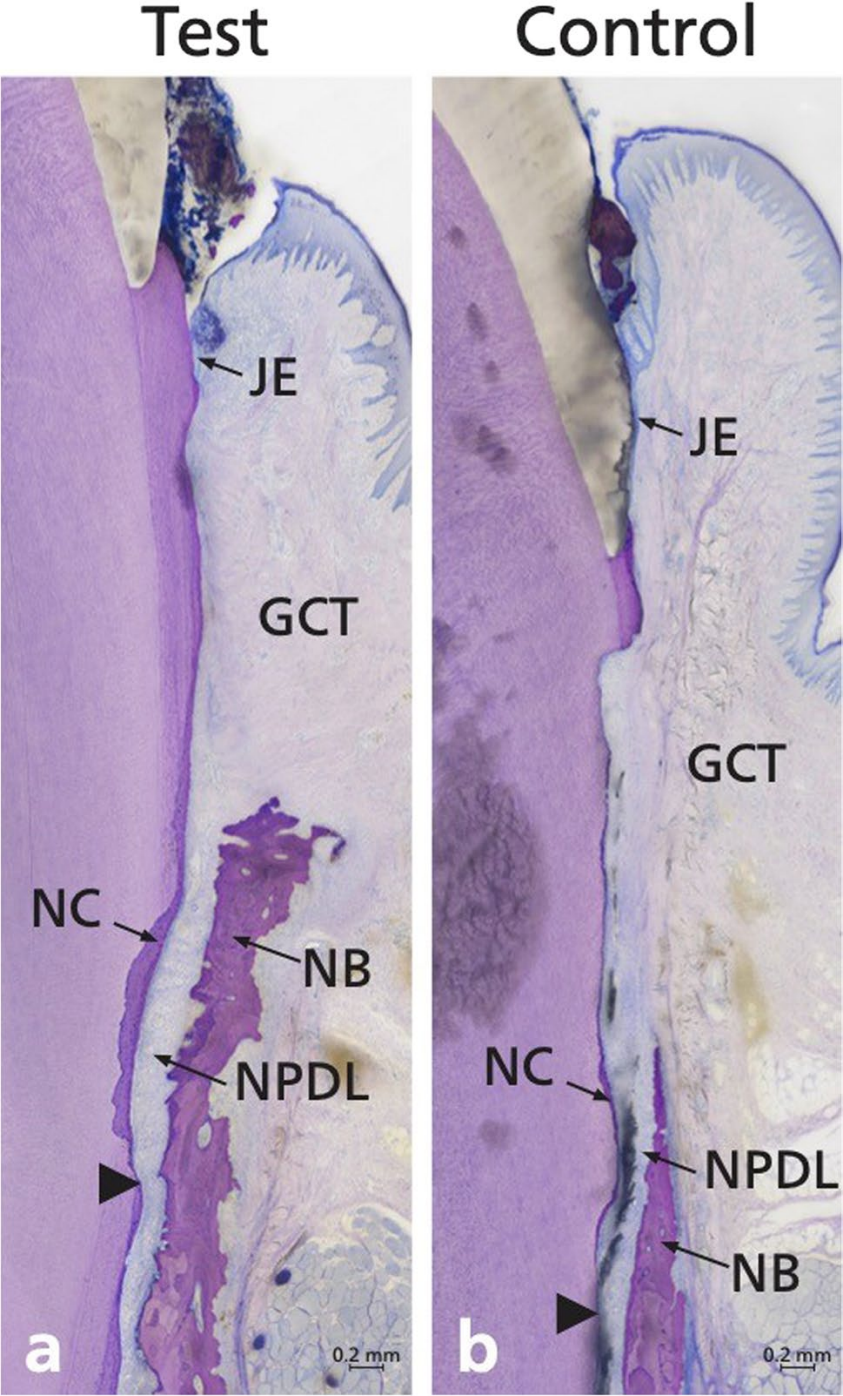
Representative overview micrographs of a **(a)** test and **(b)** control methylmethacrylate sections. Arrowheads point to the apical ends of the apical root notch. Staining: toluidine blue/McNeal + basic fuchsin. GCT, gingival connective tissue; JE, junctional epithelium; NB, new bone; NC, new cementum; NPDL, new periodontal ligament

The test (Fig. 3a) and the control group (Fig. 3b) showed varying amount of periodontal regeneration, including root cementum, periodontal ligament, and alveolar bone. The area of connective tissue adjacent to the root surface without cementum formation was broader in the control group than in the test group. The periodontal ligament fibers were in some locations already functionally oriented and in others not. The newly formed bone was, in most specimens, still immature (i.e., mainly woven bone).

### Histometric analysis

All the histometric data are shown in Table 1, including mean values, standard deviations, p-value, minimum and maximum values, median, and interquartile ranges.

**Table 1:** Histomorphometric results

	Test mean/SD	Control mean/SD	*p* value	Test min/max	Control min/max	Test median	Control median	Test 25%Perc/ 75%Perc	Control 25%Perc/ 75%Perc
Defect height	5.476 1.455	4.844 1.259	0.218	3.920 8.170	3.220 6.730	5.260	4.380	4.260 6.393	4.040 6.060
New cementum	4.377 0.359	3.477 1.127	0.046	3.960 4.910	2.020 5.390	4.290	3.540	4.157 4.840	2.290 4.120
New bone	2.148 0.795	2.239 1.226	0.937	1.030 3.523	0.700 3.920	1.980	1.910	1.740 2.770	1.420 3.910
JE + sulcus	2.240 0.818	2.654 0.517	0.296	1.467 3.680	2.110 3.410	1.860	2.470	1.480 2.850	2.220 3.280
CT adhesion	0.386 0.577	0.668 1.065	0.687	0.000 1.473	-0.930 2.100	0.030	0.690	0.010 0.890	0.050 1.900

*CT* connective tissue; *JE* junctional epithelium; *mm* millimetre; *SD* standard deviation

Defect height was 4.84 mm in the control and 5.48 mm in the test group (*p* = 0.219).

**Fig. 4:**
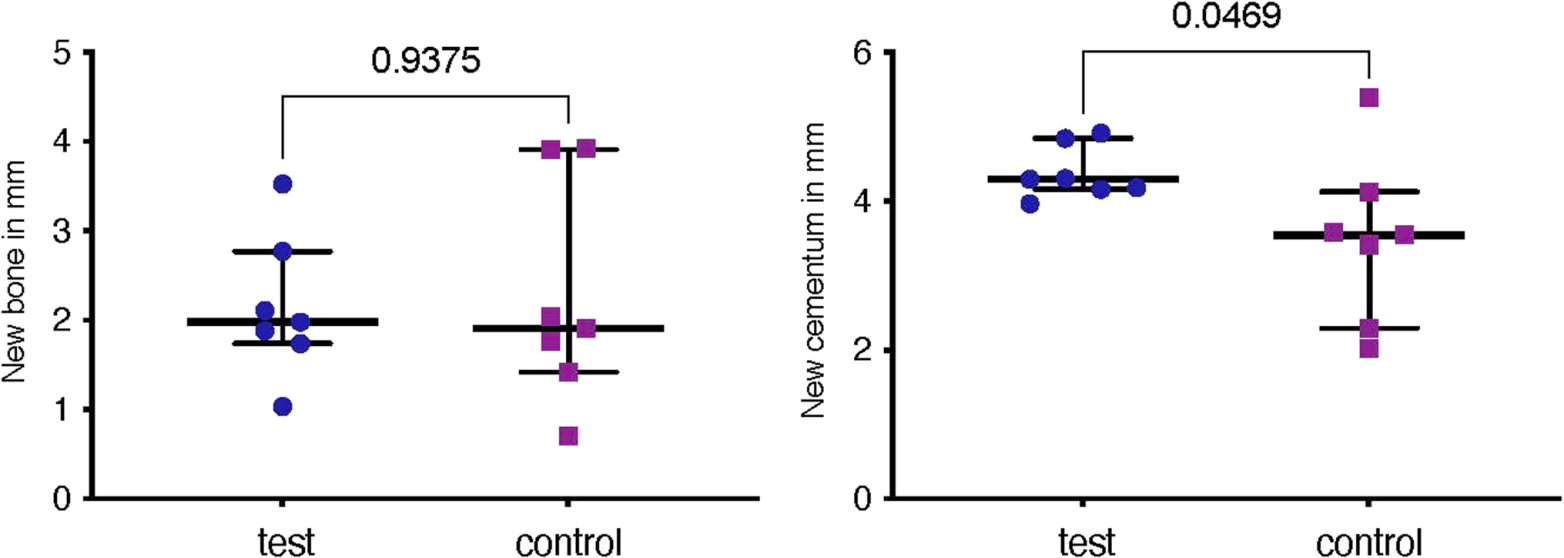
Histometric results comparing the test and control group

Cementum formation was statistically significantly higher in the test group (4.38 mm) compared to the control one (3.48 mm, *p* = 0.047). Bone formation was not statistically significantly different comparing the test (2.15 mm) and the control group (2.24 mm, *p* = 0.938). The junctional epithelium height and sulcus depth was greater in the control (2.65 mm) compared to the test group (2.24 mm), however without reaching a statistically significant difference (*p* = 0.297). The values are shown in Fig. 4.

## Discussion

The present study is the first report on the biologic potential of rAmelX on periodontal regeneration in recession-type defects, as evaluated histologically in an animal model. The results provide for the first-time evidence for regeneration of periodontal ligament and root cementum in acute-type recession defects following the application of rAmelX in conjunction with CAF. The fact that higher amounts of new cementum were measured in the test group compared to the control group points to the specific effect of rAmelX on periodontal ligament (PDL) cells, which are the key cells responsible for cementum formation. It has been repeatedly demonstrated that the main effects of EMD are exerted on PDL cells by mediating cell attachment, spreading, proliferation and survival, as well as expression of transcription factors, growth factors, cytokines, extracellular matrix constituents and other molecules involved in the regulation of cementum formation [17, 18]. Furthermore, EMD has been shown to play an essential roled in the wound healing process, promoting soft tissue regeneration and angiogenic activity [17].

However, in terms of bone regeneration there were no differences between the test and control groups. One explanation for this finding may be the use of the carrier alone in the control defects. It has been previously shown [19, 20], that in certain clinical scenarios, the use of a carrier itself may facilitate periodontal and bone regeneration by stabilizing the blood clot and the wound, preventing the flap to collapse onto the root surface and even by acting as a mechanical barrier which prevents epithelial and connective tissue cell migration into the wound.

In the present study, the control defects were treated with the thermo-sensitive poloxamer carrier. This material is characterized by changing viscosity from a liquid gel to a more viscous gel at body temperature, thereby enhancing clot formation and clot stabilization. The importance of blood clot stability and adhesion to the root surface after flap surgery has been clearly shown in previous studies [19, 21]. Thus, on one hand, it may be speculated that the control material was by itself beneficial for the regenerative result therefore masking the full regenerative potential of the tested material. On the other hand, it may be assumed that the differences in terms of cementum regeneration between the test and control groups can be attributed to the inherent biologic activity of rAmelX and not to the effect of the carrier.

Histological evidence for the effect of EMD on promoting periodontal wound healing/regeneration in recession-type defects is available from animal studies and human case reports. In 1997, Heijl [7] exhibited histologically new cementum and bone gain in one experimentally created recession defect. Rasperini et al. [8] added EMD to a subepithelial connective tissue graft and found histological evidence for new cementum, bone, and connective tissue fibers. McGuire and Cochran [6] found new cementum, organized PDL fibers, and islands of condensing bone following the use of EMD with coronally advanced flap. However, Carnio et al. [22] found this healing pattern to be inconsistent with only one of four 6-month specimens that included formation of new cementum and new bone in the most apical end of the grafted area.

Based on the high degree of sequence homology between human and porcine enamel proteins, non-erupted developing pig premolars and molars have been employed to produce EMD. Emdogain® is a commercial preparation of enamel matrix proteins composed primarily of amelogenins [23–26]. Because of the animal origin, a batch-to-batch variability can be expected. The biological effects of amelogenins are a function of the isoform/fragment employed, its concentration, implantation site, and delivery modality [27]. In vitro, its activity also depends on cell type and differentiation stage, and, in vivo, on the selected animal model [27]. Furthermore, the production of anti-EMD antibodies in the host have been also reported [13].

Thus, the use of a synthetic amelogenin peptide to avoid the use of animal-derived proteins increases safety. There is scientific evidence for the successful use of recombinant amelogenin in tissue engineering and regenerative medicine [15]. Furthermore, recombinant amelogenin showed significantly higher osteoinductive effect compared to EMD [12]. It may thus be anticipated that the use of a synthetic or recombinant peptide with a known composition increases the homogeneity, which in turn, may enhance the reproducibility of the regenerative outcome.

When interpreting the present findings, it needs to be kept in mind that the present study is the first one using this novel synthetic amelogenin product in an animal model for the treatment of recession-type defects. Due to the pilot nature of the study, the number of included animals and defects was limited and therefore, the borderline statistical significance in terms of cementum formation and the lack of statistical difference in terms of bone formation between the two groups, might be also attributed to the small sample size. Obviously, due to the different biological responses in minipigs and on the fact that the used recombinant amelogenin was X- linked human one, requires further studies to clarify the binding and release kinetics of rAmelX as well as its clinical effects in patients.

## Conclusion

Taken together, the present results have for the first time provided evidence for the potential of rAmelX to enhance regeneration of root cementum and periodontal ligament in recession-type defects, thus warranting further preclinical and clinical testing.

## Data Availability

Data are available upon request.
